# Supplementary figures and images for: Comparative evaluation of analytical methods for CSF proteomics
Source: Clin Proteomics. 2025 Nov 28;22:46. doi: 10.1186/s12014-025-09568-y (PMC12661759; doi:10.1186/s12014-025-09568-y)

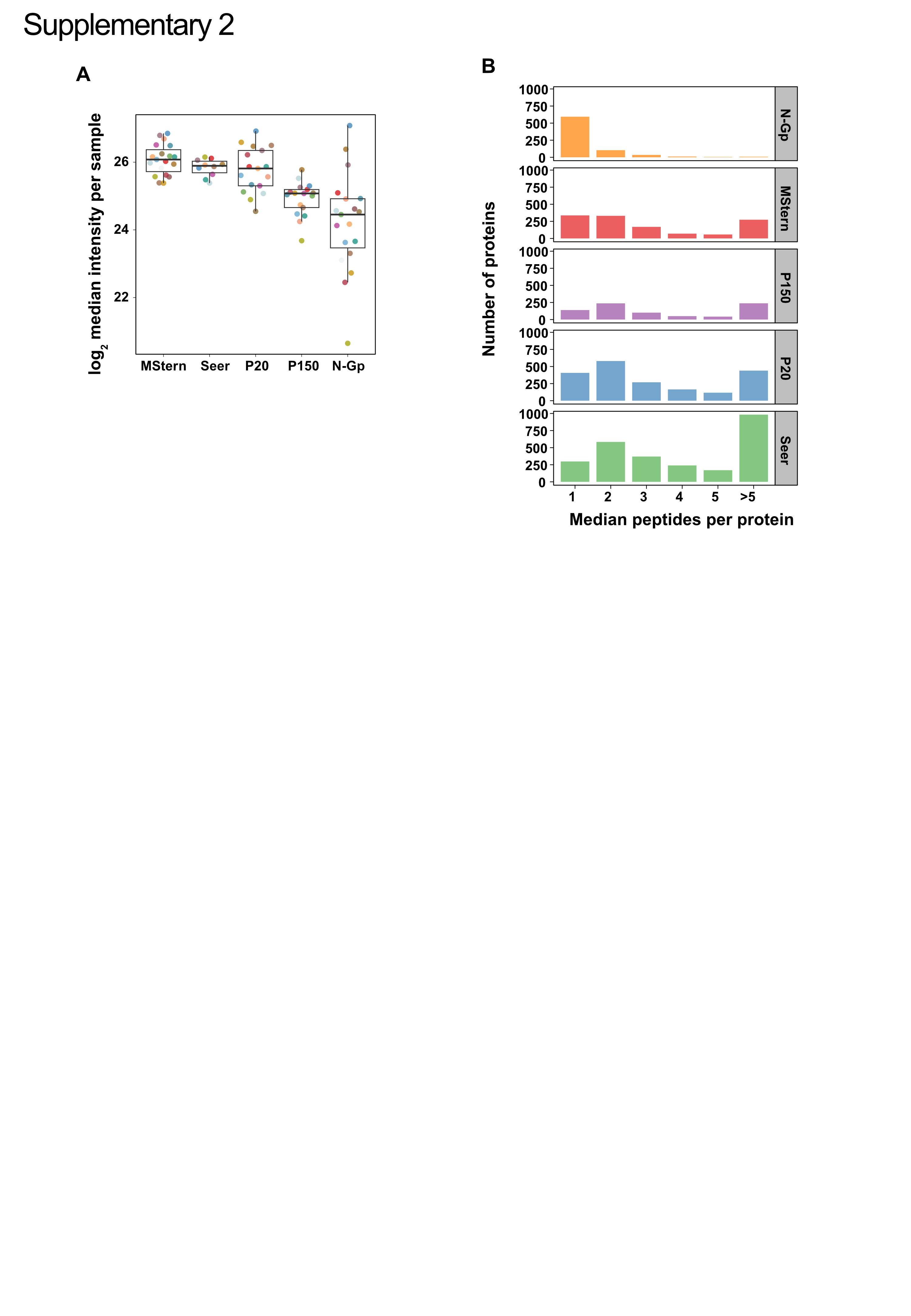

Supplement: Supplementary file 6 — Supplementary Material 6. [file 12014_2025_9568_MOESM6_ESM.tiff]

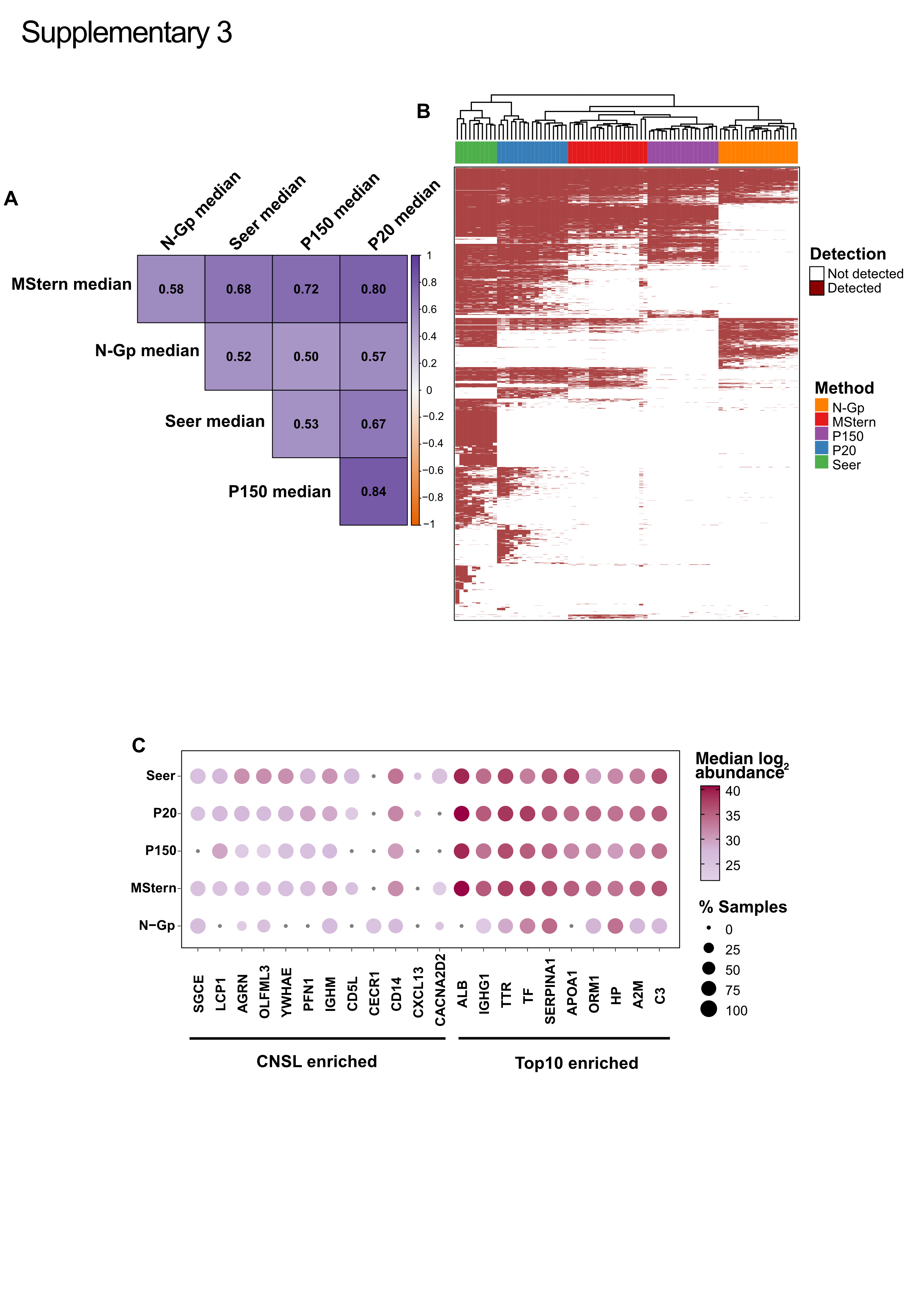

Supplement: Supplementary file 7 — Supplementary Material 7. [file 12014_2025_9568_MOESM7_ESM.tiff]

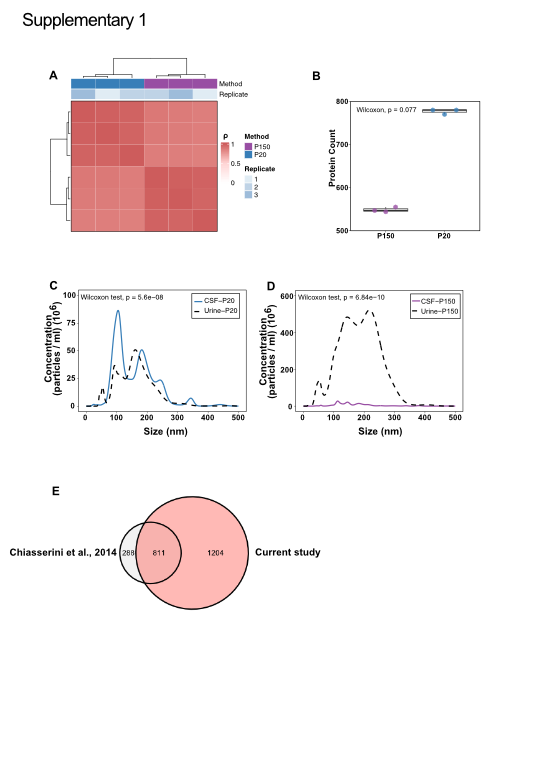

Supplement: Supplementary file 9 — Supplementary Material 9. [file 12014_2025_9568_MOESM9_ESM.tiff]
