# Supplementary material for: Comparative evaluation of analytical methods for CSF proteomics
Source: Clin Proteomics. 2025 Nov 28;22:46. doi: 10.1186/s12014-025-09568-y (PMC12661759; doi:10.1186/s12014-025-09568-y)
Supplement: Supplementary file 8 — Supplementary Material 8. [file 12014_2025_9568_MOESM8_ESM.docx]

**Supplementary Figure Legends**

**Supplementary Figure 1**

1. Clustered heatmap of pair-wise Spearman correlation coefficients for the three technical replicates generated with the P20-EV and P150-EV ultracentrifugation protocols (cohort 2).
2. Boxplot depicting the number of proteins detected per technical replicate for P150-EVs and P20-EVs in cohort 2.
3. Nanoparticle-tracking analysis of P20-EV fractions: particle-size distribution and concentration for CSF-derived EVs (solid blue line) compared with urine-derived EVs (black dashed line).
4. Equivalent analysis for P150-EV fractions (CSF, solid purple; urine, black dashed); Wilcoxon P-values for between-biofluid comparisons are indicated in each panel.
5. Venn diagram showing overlap between EV fractions in Chiasserini et al., 2014 and the current study (combined P20 and P150).

**Supplementary Figure 2**

1. Boxplot showing the median log_2_ median abundance for each method. Individual dots represent the CSF samples analysed.
2. Histograms showing the distribution of median peptide counts per protein for each workflow.

**Supplementary Figure 3**

1. Heatmap of pair-wise Spearman correlations between the per-sample median protein abundances obtained with each enrichment workflow.
2. Binary (detected / not-detected) heatmap of all protein groups, hierarchically clustered; column colours indicate the corresponding workflow.
3. Dot plot highlighting CNS-enriched markers (left panel) and the ten most abundant CSF proteins (right panel). Dot colour reflects the median log₂ intensity, whereas dot size denotes the percentage of patient samples in which the protein was identified for each workflow.

**Supplementary Data**

**Data S1.** Clinical characteristics of study participants.

**Data S2.** Mass spec data acquisition parameters.

**Data S3.** Processed proteomic profiles of CSF samples.

**Data S4.** Results from subcellular localization, related to figure 4E.

**Data S5.** Results from functional enrichment analysis, related to figure 4F.
